# The contribution of physical fitness to individual and ethnic differences in risk markers for type 2 diabetes in children: The Child Heart and Health Study in England (CHASE)

**DOI:** 10.1111/pedi.12637

**Published:** 2018-02-07

**Authors:** Claire M Nightingale, Alicja R Rudnicka, Sarah R Kerry‐Barnard, Angela S Donin, Soren Brage, Kate L Westgate, Ulf Ekelund, Derek G Cook, Christopher G Owen, Peter H Whincup

**Affiliations:** ^1^ Population Health Research Institute St George's, University of London London UK; ^2^ MRC Epidemiology Unit Institute of Metabolic Science, University of Cambridge School of Clinical Medicine Cambridge UK; ^3^ Department of Sport Medicine Norwegian School of Sport Sciences Oslo Norway

**Keywords:** children, ethnicity, physical fitness, type 2 diabetes

## Abstract

**Background:**

The relationship between physical fitness and risk markers for type 2 diabetes (T2D) in children and the contribution to ethnic differences in these risk markers have been little studied. We examined associations between physical fitness and early risk markers for T2D and cardiovascular disease in 9‐ to 10‐year‐old UK children.

**Methods:**

Cross‐sectional study of 1445 9‐ to 10‐year‐old UK children of South Asian, black African‐Caribbean and white European origin. A fasting blood sample was used for measurement of insulin, glucose (from which homeostasis model assessment [HOMA]‐insulin resistance [IR] was derived), glycated hemoglobin (HbA1c), urate, C‐reactive protein (CRP), and lipids. Measurements of blood pressure (BP) and fat mass index (FMI) were made; physical activity was measured by accelerometry. Estimated VO_2 max_ was derived from a submaximal fitness step test. Associations were estimated using multilevel linear regression.

**Results:**

Higher VO_2 max_ was associated with lower FMI, insulin, HOMA‐IR, HbA1c, glucose, urate, CRP, triglycerides, LDL‐cholesterol, BP and higher HDL‐cholesterol. Associations were reduced by adjustment for FMI, but those for insulin, HOMA‐IR, glucose, urate, CRP, triglycerides and BP remained statistically significant. Higher levels of insulin and HOMA‐IR in South Asian children were partially explained by lower levels of VO_2max_ compared to white Europeans, accounting for 11% of the difference.

**Conclusions:**

Physical fitness is associated with risk markers for T2D and CVD in children, which persist after adjustment for adiposity. Higher levels of IR in South Asians are partially explained by lower physical fitness levels compared to white Europeans. Improving physical fitness may provide scope for reducing risks of T2D.

## 1 INTRODUCTION

Higher levels of physical fitness in adults are associated with a lower risk of developing type 2 diabetes (T2D)^1^, ^2^ and lower rates of cardiovascular disease (CVD) mortality and all‐cause mortality.^3^, ^4^ South Asian adults are at higher risk of developing T2D, stroke and coronary heart disease compared to white Europeans in the UK and other western countries.^5^, ^6^ Evidence suggests that ethnic differences in risk markers for T2D (particularly insulin resistance) are apparent in prepubertal children with higher levels of T2D risk markers in South Asian children compared to white Europeans.^7^ Ethnic differences in physical fitness are also apparent in childhood; we have shown in a recent report lower levels of physical fitness in South Asians compared to white Europeans.^8^ These differences in physical fitness could potentially help to explain ethnic differences in risk markers for T2D. We therefore examined associations between physical fitness and risk markers for T2D and CVD in a study of British school children and examined the contribution of differences in physical fitness to ethnic differences in risk markers for T2D between South Asians and white Europeans. It has been suggested that physical fitness and physical activity could have separate and independent effects on metabolic risk in childhood.^9^ Given the complex relationship between physical activity and fitness, we also examine the independent associations between physical activity, physical fitness, and risk for T2D and CVD.

## 2 METHODS

The Child Heart and Health Study in England (CHASE) was a cross‐sectional study of the health of 9‐ to 10‐year‐old children of white European, South Asian and black African‐Caribbean origin carried out between 2004 and 2007 in London, Birmingham and Leicester. Ethical approval was obtained from the relevant multicenter research ethics committee and informed, written consent was obtained from the parents of all participating children. Full details have been published elsewhere 7, 10, 11. In brief, the study was based on a random sample of 200 state primary schools, half were drawn from a sampling frame of schools with a high prevalence of UK South Asian children and half were drawn from a sampling frame with a high prevalence of UK black African‐Caribbean children. This report is based on the final phase of the study carried out between January 2006 and February 2007 in which children from 81 schools had assessments of physical fitness and physical activity.

A single survey team consisting of 3 trained Research Nurses and 2 Research Assistants carried out all assessments in schools. Physical measurements included height, weight, and arm‐to‐leg bioelectrical impedance, using the Bodystat 1500 monitor (Bodystat Ltd, Douglas, Isle of Man, UK). Fat mass was derived from bioelectrical impedance using gender‐ and ethnic‐specific equations derived for children of this age group.^12^ Fat mass index (FMI, fat mass [kg]/height [m]^5^) was derived to be uncorrelated with height (*r* = −0.04) which has been shown previously to provide a more valid measure of body fatness in this multi‐ethnic study population.^13^ Systolic and diastolic blood pressure (BP) were measured twice in the right arm using an Omron HEM‐907 (Omron Electronics, Milton Keynes, UK). Mean systolic and diastolic BP were adjusted for cuff size using a previously validated method.^14^

Participants provided a blood sample after an overnight fast; children who reported having eaten breakfast were excluded from the analysis. Breakfast was provided after the sample was collected. Serum was separated and frozen on dry ice immediately after collection for measurement of insulin. Urate was measured in serum using an enzymatic assay.^15^ Samples were shipped to a central laboratory within 48 hours of collection. Details of insulin, glucose, glycated hemoglobin (HbA1c), C‐reactive protein (CRP) and blood lipids assays have been previously reported.^7^, ^10^ Homeostasis model assessment (HOMA) equations^16^ were used to derive insulin resistance from fasting insulin and glucose measurements.

An 8‐minute submaximal step test was performed by participating children as previously described.^17^, ^18^ In brief, participants followed an audible prompt which instructed them to increase their step frequency progressively from 15 to 32.5 body lifts/min on a 150‐mm high step while wearing a combined heart rate (HR) and movement sensor (Actiheart, CamNtech, Papworth, UK). The test was terminated if the participant was unable to sustain the prescribed step frequency. At the end of the test, 2 minutes of seated recovery was recorded. ECG and acceleration waveforms were recorded at 128 and 32 Hz sampling, respectively. VO_2 max_ was estimated using a similar method to that used in the Health Survey for England 2008.^19^ In brief, predicted workload was regressed against instantaneous HR (expressed above resting HR) and 1‐minute recovery HR was extracted using quadratic regression against recovery time. This was combined with resting HR and test duration to calculate the submaximal relationship between workload and HR, which was extrapolated to predicted maximal HR to predict maximal work capacity. This result was converted to VO_2 max_ by adding an estimate of resting metabolic rate and then dividing by the energetic value of oxygen.^19^

Objective measures of physical activity were made using an accelerometer recorded at 5 second epochs (GT1M; ActiGraph LLC, Pensacola, Florida), worn on the participant's left hip during waking hours for 7 days; the device was removed for water‐based activities. Non‐wear time was defined as a period of at least 20 consecutive minutes of zero counts, and was excluded from the analyses. Physical activity was summarized as total daily counts. Participants with at least 1 day of valid data (defined as at least 600 minutes of registered time) were included in the analysis.

Ethnic origin was defined using information from a parental questionnaire on the ethnicity of both parents where available (63%), or using the parentally defined ethnic origin of the child (36%). In a small number of children (1%) where this information was not available, ethnic origin was defined using information on parental and grandparental place of birth provided by the child, cross‐checked with observer assessment of ethnic origin. Children were defined as white European, South Asian, black African‐Caribbean and “other” ethnicity.

### 2.1 Statistical methods

Statistical analyses were carried out using Stata/SE software (Stata/SE 13 for Windows; StataCorp LP, College Station, Texas). All risk markers were inspected for normality; all variables except systolic and diastolic BP followed a log‐normal distribution and were therefore log‐transformed in analyses. A regression calibration method to allow for measurement error in physical activity counts^20^ was used to allow for within‐child variation by day of the week and variation in the number of days of recording (between 1 and 7), which provides an unbiased average for each child. The majority of participants (83%) wore the ActiGraph for at least 4 days. We examined the shape of associations between VO_2 max_ and risk markers by deciles of VO_2 max_ and did not find any evidence of departures from linearity. Hence, associations between estimated VO_2 max_ and risk markers were assessed using multilevel linear models adjusted for sex, age (in quartiles), ethnic group, month of measurement and height (fitted as fixed effects) and school fitted as a random effect to take into account clustering of children within schools. The effect of additional adjustment for FMI was also examined. We also examined whether associations between VO_2 max_ and risk markers were consistent in boys and girls, and ethnic groups. To elucidate whether ethnic differences in estimated risk markers were explained by differences in physical fitness, we examined the effect of adjustment for VO_2 max_ on ethnic differences in risk markers. In separate models we investigated whether associations between physical fitness and risk markers were consistent across tertiles of physical activity.

## 3 RESULTS

The participation rate for children taking part in this phase of the study was 63%; of those who took part 1979 (89%) provided a fasting blood sample and had complete data. Estimated VO_2 max_ values were available for a total of 1445 participants (50% female with a mean age of 10.0 years [95% reference range 9.2, 10.7 years]) who had provided a fasting blood sample and had measurements of FMI. Of these children, 1083 provided a valid estimate of physical activity. The reasons for missing physical activity data were refusal to wear the device, failure to return the device or providing less than 1 day with 600 minutes of wear time of the device. Adjusted mean levels of VO_2 max_ and risk markers for T2D and CVD are shown in Table 1. Boys had higher estimated levels of VO_2 max_ than girls; South Asians had lower levels and black African‐Caribbeans had higher levels of estimated VO_2 max_ compared to white Europeans.

**Table 1:** Estimated VO_2 max_ and risk markers for type 2 diabetes and cardiovascular disease by sex and ethnic group

	Geometric mean/mean^a^ (95% CI)												
	Boys (*n* = 723)		Girls (*n* = 722)		White European (*n* = 389)		South Asian (*n* = 373)		Black African‐Caribbean (*n* = 346)		Other (*n* = 337)		*P*‐value (ethnic group)^b^
Fat mass index (kg/m^5^)	2.0	(1.9, 2.0)	2.2	(2.1, 2.3)	2.1	(2.0, 2.2)	2.1	(2.1, 2.2)	1.9	(1.8, 2.0)	2.2	(2.1, 2.3)	<.0001
Estimated VO_2 max_ (mL O_2_/min/kg)^a^	40.8	(40.4, 41.2)	37.9	(37.5, 38.3)	39.3	(38.9, 39.8)	38.5	(37.9, 39.0)	40.3	(39.8, 40.8)	39.3	(38.8, 39.8)	<.0001
Physical activity counts (×1000)^a^, ^c^	437	(428, 445)	364	(356, 373)	400	(390, 410)	378	(367, 390)	422	(411, 432)	398	(387, 408)	<.0001
Insulin (mU/L)	6.53	(6.16, 6.92)	8.05	(7.59, 8.53)	6.26	(5.84, 6.72)	8.68	(8.03, 9.39)	6.64	(6.16, 7.16)	7.69	(7.14, 8.28)	<.0001
HOMA‐insulin resistance	0.82	(0.78, 0.87)	1.00	(0.94, 1.06)	0.78	(0.73, 0.83)	1.08	(1.01, 1.17)	0.83	(0.77, 0.90)	0.96	(0.89, 1.03)	<.0001
HbA1c (%)	5.29	(5.26, 5.31)	5.29	(5.26, 5.31)	5.23	(5.20, 5.26)	5.33	(5.29, 5.37)	5.30	(5.27, 5.34)	5.29	(5.25, 5.32)	<.001
HbA1c (mmol/mol)	34.0	(33.8, 34.3)	34.1	(33.8, 34.4)	33.5	(33.1, 33.8)	34.5	(34.1, 34.9)	34.2	(33.8, 34.6)	34.1	(33.7, 34.5)	<.001
Glucose (mmol/L)	4.51	(4.49, 4.54)	4.43	(4.40, 4.45)	4.45	(4.41, 4.48)	4.51	(4.47, 4.55)	4.43	(4.39, 4.46)	4.49	(4.46, 4.53)	.002
Urate (mmol/L)	0.22	(0.21, 0.22)	0.23	(0.22, 0.23)	0.22	(0.22, 0.23)	0.23	(0.22, 0.23)	0.21	(0.20, 0.21)	0.23	(0.22, 0.23)	<.0001
C‐reactive protein (mg/L)	0.45	(0.41, 0.49)	0.60	(0.54, 0.65)	0.48	(0.43, 0.55)	0.58	(0.51, 0.67)	0.46	(0.40, 0.53)	0.55	(0.48, 0.63)	.06
Triglyceride (mmol/L)	0.80	(0.78, 0.83)	0.89	(0.86, 0.92)	0.83	(0.80, 0.86)	0.96	(0.92, 1.00)	0.73	(0.70, 0.76)	0.87	(0.84, 0.91)	<.0001
HDL‐cholesterol (mmol/L)	1.53	(1.51, 1.56)	1.46	(1.44, 1.48)	1.51	(1.48, 1.54)	1.44	(1.41, 1.48)	1.55	(1.51, 1.59)	1.48	(1.45, 1.52)	<.001
LDL‐cholesterol (mmol/L)	2.55	(2.50, 2.60)	2.52	(2.48, 2.57)	2.52	(2.45, 2.58)	2.56	(2.49, 2.63)	2.50	(2.44, 2.58)	2.56	(2.49, 2.63)	.56
Systolic blood pressure (mm Hg)^a^	105.2	(104.3, 106.2)	103.6	(102.7, 104.6)	105.1	(103.9, 106.3)	104.6	(103.3, 105.9)	102.5	(101.2, 103.8)	105.5	(104.2, 106.7)	<.001
Diastolic blood pressure (mm Hg)^a^	62.9	(62.1, 63.7)	63.3	(62.5, 64.1)	62.6	(61.6, 63.6)	63.8	(62.6, 64.9)	62.7	(61.6, 63.7)	63.4	(62.4, 64.5)	.32

Geometric means are shown for all variables which are log‐transformed.

Means presented by sex are adjusted for age (in quartiles), ethnic group, month of measurement height, and school (random effect).

Means presented by ethnic group are adjusted for sex, age (in quartiles), month of measurement height, and school (random effect).

a Means are shown for untransformed variables.

b *P*‐value for heterogeneity in association with ethnic group.

c *N* = 1083 for physical activity counts.

Associations between physical fitness (estimated VO_2 max_) and risk markers for T2D and CVD are shown in Table 2. Estimated VO_2 max_ was inversely associated with FMI, fasting insulin, HOMA‐insulin resistance (IR), HbA1c, fasting glucose, urate, CRP, triglyceride, LDL‐cholesterol, and systolic and diastolic BP, and positively associated with HDL‐cholesterol. Associations between estimated VO_2 max_ and insulin, HOMA‐IR, glucose, urate, CRP and triglyceride were attenuated by adjustment for FMI between 32% and 63%. Further adjustment for fat free mass index (FFMI) did not further attenuate these associations (data available from authors). Associations with HbA1c, HDL‐ and LDL‐cholesterol were completely explained by adjustment for FMI and those with systolic and diastolic BP were attenuated by 31% and 16%, respectively. After adjustment for FMI, a 1 IQR increase in estimated VO_2 max_ was associated with lower fasting insulin, HOMA‐IR, fasting glucose, urate, CRP, and triglyceride of 7.7%, 7.7%, 0.7%, 2.2%, 20.8%, and 5.3%, respectively. Similarly, a 1 IQR increase in estimated VO_2 max_ was associated with decreases in systolic and diastolic BP of 1.7 and 2.7 mm Hg, respectively. These associations were broadly similar in boys and girls (Table S1) and in South Asians, black African‐Caribbeans, white Europeans and “other” ethnic groups (Table S2) with the exception of LDL‐cholesterol which was inversely associated with VO_2 max_ in all ethnic groups except black African‐Caribbeans.

**Table 2:** Associations between estimated VO_2 max_ and risk markers for type 2 diabetes and cardiovascular disease with additional adjustment for fat mass index (FMI)

Risk markers (*N* = 1445)	Adjustments	Percentage difference/difference^a^ (95% CI) for 1 IQR increase in estimated VO_2 max_		*P*‐value
FMI (kg/m^5^)	Standard	−16.28	(−18.44, −14.06)	<.0001
Insulin (mU/L)	Standard	−18.87	(−22.27, −15.33)	<.0001
	Standard + FMI	−7.69	(−11.37, −3.85)	<.001
HOMA‐insulin resistance	Standard	−18.80	(−22.22, −15.23)	<.0001
	Standard + FMI	−7.68	(−11.40, −3.80)	<.001
HbA1c (%)	Standard	−0.56	(−1.02, −0.10)	.02
	Standard + FMI	−0.34	(−0.83, 0.15)	.17
Glucose (mmol/L)	Standard	−0.99	(−1.53, −0.45)	<.001
	Standard + FMI	−0.67	(−1.24, −0.09)	.02
Urate (mmol/L)	Standard	−5.85	(−7.56, −4.10)	<.0001
	Standard + FMI	−2.19	(−3.99, −0.35)	.02
C‐reactive protein (mg/L)	Standard	−40.85	(−46.11, −35.06)	<.0001
	Standard + FMI	−20.84	(−27.52, −13.54)	<.0001
Triglyceride (mmol/L)	Standard	−9.79	(−12.27, −7.24)	<.0001
	Standard + FMI	−5.26	(−7.93, −2.51)	<.001
HDL‐cholesterol (mmol/L)	Standard	3.78	(2.18, 5.41)	<.0001
	Standard + FMI	0.78	(−0.81, 2.39)	.34
LDL‐cholesterol (mmol/L)	Standard	−3.47	(−5.34, −1.57)	<.001
	Standard + FMI	−1.80	(−3.80, 0.24)	.08
Systolic blood pressure (mm Hg)^a^	Standard	−2.46	(−3.22, −1.70)	<.0001
	Standard + FMI	−1.69	(−2.48, −0.89)	<.0001
Diastolic blood pressure (mm Hg)^a^	Standard	−3.17	(−3.86, −2.49)	<.0001
	Standard + FMI	−2.66	(−3.38, −1.94)	<.0001

Percentage differences are shown for all variables which are log‐transformed.

Standard adjustment is for sex, age (in quartiles), ethnic group, month of measurement, height, and school (random effect).

a Absolute differences are shown for untransformed variables.

In a subset of 1083 children with objective measures of both physical fitness and physical activity, associations between risk markers for T2D/CVD and physical activity and VO_2 max_ are shown in Table S3. Associations were stronger between risk markers for T2D and estimated VO_2 max_ compared to those for overall physical activity. Following adjustment for FMI, however, associations between fasting insulin, HOMA‐IR and physical activity were stronger than those for estimated VO_2 max_. To explore whether the associations between physical fitness and risk markers were modified by physical activity level, associations between VO_2 max_ and risk markers were examined by tertiles of physical activity in Table S4. The inverse associations between estimated VO_2 max_ and fasting insulin, HOMA‐IR and triglyceride were strongest among children in the lowest tertile of physical activity and weakest among children in the highest tertile; a graphical representation for fasting insulin is shown in Figure S1.

Ethnic differences in risk markers for T2D between South Asians and white Europeans and the effect of adjustment for physical fitness are shown in Table 3. South Asians had higher levels of fasting insulin, HOMA‐IR, HbA1c, fasting glucose and triglyceride, and lower levels of HDL‐cholesterol. Adjustment for VO_2 max_ reduced these ethnic differences in insulin, HOMA‐IR, glucose and triglyceride by approximately 11%; differences in HbA1c and HDL‐cholesterol were reduced by 4% and 13%, respectively. The borderline ethnic difference in CRP was reduced by 50% by adjustment for estimated VO_2 max_. In the subset of 1083 participants with measurements of physical activity, Table S5 shows whether adjustment for physical activity counts in addition to VO_2 max_ would further explain the ethnic differences in risk markers for T2D. For fasting insulin, HOMA‐IR, triglycerides and HDL‐cholesterol adjustment for physical activity as well as VO_2 max_, further reduced the difference between South Asians and white Europeans by 5% to 7%.

**Table 3:** Ethnic differences in risk markers for type 2 diabetes and cardiovascular disease: effect of adjustment for estimated VO_2 max_

		Percentage difference/difference^a^ (95% CI), *P*‐value, % reduction in ethnic difference following adjustment for estimated VO_2 max_			
Risk markers (*N* = 1445)	Adjustments	South Asian‐white European			
Insulin (mU/L)	Standard	38.63	(26.78, 51.59)	<.0001	
	Standard + VO_2 max_	34.52	(23.32, 46.73)	<.0001	10.6
HOMA‐insulin resistance	Standard	39.34	(27.41, 52.39)	<.0001	
	Standard + VO_2 max_	35.19	(23.90, 47.50)	<.0001	10.5
HbA1c (%)	Standard	1.93	(1.00, 2.86)	<.0001	
	Standard + VO_2 max_	1.85	(0.92, 2.78)	<.0001	4.1
Glucose (mmol/L)	Standard	1.41	(0.31, 2.51)	.01	
	Standard + VO_2 max_	1.25	(0.16, 2.36)	.02	11.3
Urate (mmol/L)	Standard	1.76	(−1.91, 5.57)	.35	
	Standard + VO_2 max_	0.86	(−2.72, 4.58)	.64	51.1
C‐reactive protein (mg/L)	Standard	20.80	(0.21, 45.63)	.05	
	Standard + VO_2 max_	10.37	(−8.09, 32.54)	.29	50.1
Triglyceride (mmol/L)	Standard	15.63	(9.27, 22.36)	<.0001	
	Standard + VO_2 max_	13.92	(7.74, 20.44)	<.0001	10.9
HDL‐cholesterol (mmol/L)	Standard	−4.27	(−7.20, −1.24)	.01	
	Standard + VO_2 max_	−3.72	(−6.66, −0.68)	.02	12.9
LDL‐cholesterol (mmol/L)	Standard	1.87	(−1.92, 5.80)	.34	
	Standard + VO_2 max_	1.31	(−2.45, 5.21)	.50	29.9
Systolic blood pressure (mm Hg)^a^	Standard	−0.47	(−2.02, 1.09)	.56	
	Standard + VO_2 max_	−0.83	(−2.37, 0.71)	.29	−76.6
Diastolic blood pressure (mm Hg)^a^	Standard	1.19	(−0.21, 2.59)	.10	
	Standard + VO_2 max_	0.76	(−0.61, 2.13)	.28	36.1

Percentage differences are shown for all variables which are log‐transformed.

Standard adjustment is for sex, age (in quartiles), ethnic group, month of measurement, height, and school (random effect).

a Absolute differences are shown for untransformed variables.

## 4 DISCUSSION

### 4.1 Main findings

This study provides strong evidence that low levels of physical fitness are associated with risk markers for T2D and CVD in this multi‐ethnic population of school children. These associations were attenuated but remained statistically significant after adjustment for measures of body fat, suggesting that associations were at least partly reduced by the effect of adjustment for adiposity, though additional adjustment for fat free mass had no material effect. Moreover, ethnic differences in risk markers for T2D, particularly increased levels of insulin resistance in South Asians compared to white European children, were partially explained by physical fitness (approximately 11%). Stratified analysis suggested that the association between physical fitness and risk markers differed by level of physical activity, with stronger associations being observed at lower levels of physical activity.

### 4.2 Relation to previous studies

The finding in the present study that objective measures of physical fitness are inversely associated with risk markers for T2D and CVD in childhood is consistent with a small number of studies, which have used similar objective methodologies.^21^ In particular, findings from the European Youth Heart Study (EYHS) showed that maximal ergometer assessments of cardiorespiratory fitness were inversely related to metabolic risk markers at both 9 and 15 years of age.^21^, ^22^ These associations have since been demonstrated in prospective studies, providing further evidence of a causal relationship.^23^ We have previously reported that both physical activity and adiposity are associated with risk markers for T2D and CVD in this study population,^20^, ^24^ and that these associations are partially, but not wholly, mutually independent.

The present study confirms the association between physical fitness and cardiometabolic risk markers, but is novel in suggesting that the association observed might be altered at different levels of physical activity, with stronger associations between physical fitness and metabolic risk at lower levels of physical activity. This is consistent with a report based on 589 Danish children in the EYHS but this was not confirmed in the full EYHS study population, which suggested little or no modifying influence of physical activity.^21^ Intuitively it would seem plausible that physical fitness‐metabolic risk associations might be modified by levels of physical activity, given the established link between activity and fitness, where previous physical activity interventions have been shown to improve physical fitness.^25^, ^26^, ^27^ However, this apparent interaction could also reflect differences in the precision of measurement of physical activity and physical fitness, and the possibility of non‐linear associations between them. A sensitivity analysis examining these associations in children with at least 6 days of physical activity data (54% of the sample) showed that stronger associations between risk markers and physical fitness persisted at lower levels of physical activity (data available from authors).

This study suggests that adiposity is an important potential mediator of the associations between physical fitness and risk markers for T2D and CVD. Adjustment for objective markers of body fatness (FMI), reduces fitness‐metabolic associations between one‐third and two‐thirds, and weakens associations with some cardiovascular risk markers even further. Previous studies have been inconsistent about the confounding influence of adiposity,^21^, ^28^, ^29^, ^30^ and whether adiposity confounds, mediates or modifies associations between fitness and metabolic risk markers in early life. Randomized controlled trials are needed to further elaborate on the causal nature of these associations. Teasing out the potential interrelationship between these factors may have ramifications for future interventions, which may need to target improvements in both levels of physical activity and fitness, as well as adiposity, in order to improve metabolic and cardiovascular health from an early age.

Previous studies examining the consistency of early physical fitness and T2D/CVD risk marker associations have suggested that these are similar in boys and girls, and show little regional variations among populations of European ancestry.^31^ The present study confirms that associations were similar by sex, and extends knowledge by showing consistent associations in children of more diverse ethnic origin, including those of white European, South Asian, and black African‐Caribbean origin. As far as we are aware, the current study is also novel in showing that early ethnic differences in risk markers for T2D, where those of South Asian origin have a worse metabolic profile^32^ can be at least partly “explained” by levels of physical fitness. Risk markers for T2D in black African‐Caribbean children showed smaller differences from white Europeans, and physical fitness levels were higher compared to white Europeans suggesting this would not help to explain any of the difference.

### 4.3 Strengths and limitations

There are a number of strengths and weaknesses worthy of consideration. A strength of the study is the objective measure of physical fitness using a submaximal fitness test to estimate VO_2 max,_ 8 which has previously been used in a nationally representative large‐scale study including this age group.^19^ The use of a submaximal fitness test means that VO_2 max_ must be extrapolated, however, this test was chosen for its suitability in large‐scale studies^19^ and to encourage participation among a target population with low levels of physical activity.^11^ This was accompanied by objective assessment of physical activity, using waist worn accelerometry over a 7‐day period, which provides a reliable assessment of habitual levels of physical activity.^20^ While both these measures provide accurate assessment, their comparative accuracy should be considered. The former provides a single measure of a long‐term attribute (indicative of fitness levels over a month or longer), whereas accelerometry provides a measure of a short‐term behavior, which may not fully capture habitual levels of physical activity behavior. Hence, while the fitness‐metabolic or cardiovascular risk marker associations might be considered robust, the predictive value of physical activity may have been underestimated. Specifically fitness is partly a function of long‐term sustained physical activity, so it may be representing effects of past activity and/or more intense activity.

Another key strength is the measurement of important early risk markers for T2D (including HOMA insulin resistance, HbA1c, triglyceride, and urate) and CVD (including systolic and diastolic BP and LDL‐cholesterol). In addition, adiposity was measured using bioelectrical impedance with ethnic‐ and gender‐specific equations for the prediction of fat mass in this age group; this method has been shown to provide valid assessments of adiposity in a multi‐ethnic population.^12^ While the response rate for the present study was modest, the sample contained balanced representation of South Asians, black African‐Caribbeans and white Europeans, and of boys and girls. Furthermore, risk markers were similar in those who completed the fitness test and those who did not (data not presented). Ethnic differences in metabolic risk markers in the CHASE study have previously been reported in the whole study population^32^; patterns of ethnic differences were broadly similar in this subset of data.

### 4.4 Implications

The results presented in the present study show that ethnic differences in physical fitness account for up to 11% of the difference in insulin resistance and glycaemia markers between South Asians (who had higher levels of these markers) and white Europeans. This has potential implications for the early prevention of T2D in South Asians. Clinical trials are needed to determine whether efforts to improve physical fitness (potentially through increases in vigorous intensity physical activity) could help to improve metabolic health in children, perhaps especially among South Asian children/adolescents in whom risks of insulin resistance and T2D are particularly high.^5^, ^6^

## Supporting information

Table S1. Associations between estimated VO_2 max_ and risk markers for type 2 diabetes and cardiovascular disease (differences per one unit increase in estimated VO_2 max_ in mL O_2_/min/kg) with adjustments for fat mass index: by sex.
Table S2. Associations between estimated VO_2 max_ and risk markers for type 2 diabetes and cardiovascular disease (differences per one unit increase in estimated VO_2 max_ in mL O_2_/min/kg) with adjustments for fat mass index: by ethnic group.
Table S3. Associations between estimated VO_2 max_, physical activity and risk markers for type 2 diabetes and cardiovascular disease (differences per 1 IQR increase in estimated VO_2 max_ or counts), with adjustment for fat mass index.
Table S4. Associations between estimated VO_2 max_ and risk markers for type 2 diabetes and cardiovascular disease (differences per 1 IQR increase in estimated VO_2 max_) by tertiles of physical activity counts.
Table S5. Ethnic differences in risk markers for type 2 diabetes and cardiovascular disease: effect of adjustment for estimated VO_2 max_ and physical activity counts.
Figure S1. Mean fasting insulin by tertiles of physical activity and physical fitness.
