# Supplementary material for: The contribution of physical fitness to individual and ethnic differences in risk markers for type 2 diabetes in children: The Child Heart and Health Study in England (CHASE)
Source: Pediatr Diabetes. 2018 Feb 7;19(4):603–10. doi: 10.1111/pedi.12637 (PMC5969256; doi:10.1111/pedi.12637)
Supplement: Supplementary file 1 — Table S1. Associations between estimated VO2 max and risk markers for type 2 diabetes and cardiovascular disease (differences per one unit increase in estimated VO2 max in mL O2/min/kg) with adjustments for fat mass index: by sex. Table S2. Associations between estimated VO2 max and risk markers for type 2 diabetes and cardiovascular disease (differences per one unit increase in estimated VO2 max in mL O2/min/kg) with adjustments for fat mass index: by ethnic group. Table S3. Associations between estimated VO2 max, physical activity and risk markers for type 2 diabetes and cardiovascular disease (differences per 1 IQR increase in estimated VO2 max or counts), with adjustment for fat mass index. Table S4. Associations between estimated VO2 max and risk markers for type 2 diabetes and cardiovascular disease (differences per 1 IQR increase in estimated VO2 max) by tertiles of physical activity counts. Table S5. Ethnic differences in risk markers for type 2 diabetes and cardiovascular disease: effect of adjustment for estimated VO2 max and physical activity counts. Figure S1. Mean fasting insulin by tertiles of physical activity and physical fitness. [file PEDI-19-603-s001.docx]

Supplementary Table 1: Associations between estimated VO_2 max_ and risk markers for type 2 diabetes and cardiovascular disease (differences per one unit increase in estimated VO_2 max_ in ml O_2_/min/kg) with adjustments for fat mass index: by sex

| Risk markers (N = 1445) | Adjustments | % difference/difference* (95% CI) for a one IQR increase in estimated VO_2 max_ | | | | p(sex difference in association) |
| --- | --- | --- | --- | --- | --- | --- |
|  |  | Boys (n=723) | | Girls (n=722) | |  |
| Insulin (mU/l) | Standard | -17.99 | (-22.54, -13.18) | -19.87 | (-24.61, -14.84) | 0.58 |
|  | Standard + FMI | -6.04 | (-10.91, -0.90) | -9.52 | (-14.46, -4.30) | 0.31 |
| HOMA Insulin resistance | Standard | -17.79 | (-22.38, -12.93) | -19.94 | (-24.71, -14.88) | 0.53 |
|  | Standard + FMI | -5.88 | (-10.81, -0.68) | -9.67 | (-14.66, -4.39) | 0.27 |
| HbA1c (%) | Standard | -0.87 | (-1.49, -0.25) | -0.20 | (-0.87, 0.46) | 0.14 |
|  | Standard + FMI | -0.64 | (-1.28, 0.00) | 0.00 | (-0.68, 0.69) | 0.16 |
| Glucose (mmol/l) | Standard | -1.23 | (-1.95, -0.50) | -0.72 | (-1.49, 0.07) | 0.34 |
|  | Standard + FMI | -0.89 | (-1.64, -0.13) | -0.42 | (-1.21, 0.39) | 0.38 |
| Urate (mmol/l) | Standard | -6.18 | (-8.46, -3.84) | -5.46 | (-7.92, -2.94) | 0.68 |
|  | Standard + FMI | -2.33 | (-4.69, 0.09) | -2.03 | (-4.53, 0.54) | 0.87 |
| C-reactive protein (mg/l) | Standard | -40.33 | (-47.37, -32.36) | -41.42 | (-48.78, -33.01) | 0.83 |
|  | Standard + FMI | -18.83 | (-27.70, -8.87) | -23.04 | (-31.90, -13.03) | 0.49 |
| Triglyceride (mmol/l) | Standard | -11.93 | (-15.16, -8.59) | -7.26 | (-10.88, -3.49) | 0.05 |
|  | Standard + FMI | -7.27 | (-10.68, -3.73) | -2.92 | (-6.69, 1.00) | 0.07 |
| HDL cholesterol (mmol/l) | Standard | 3.80 | (1.66, 5.99) | 3.76 | (1.47, 6.10) | 0.94 |
|  | Standard + FMI | 0.62 | (-1.46, 2.74) | 0.95 | (-1.25, 3.20) | 0.85 |
| LDL cholesterol (mmol/l) | Standard | -4.04 | (-6.53, -1.48) | -2.82 | (-5.51, -0.04) | 0.51 |
|  | Standard + FMI | -2.28 | (-4.90, 0.41) | -1.26 | (-4.05, 1.61) | 0.59 |
| Systolic blood pressure (mmHg)* | Standard | -2.47 | (-3.49, -1.46) | -2.44 | (-3.52, -1.35) | 0.97 |
|  | Standard + FMI | -1.66 | (-2.70, -0.62) | -1.71 | (-2.82, -0.61) | 0.93 |
| Diastolic blood pressure (mmHg)* | Standard | -2.64 | (-3.55, -1.73) | -3.79 | (-4.76, -2.81) | 0.09 |
|  | Standard + FMI | -2.10 | (-3.04, -1.15) | -3.30 | (-4.30, -2.31) | 0.07 |

* Absolute differences are shown for untransformed variables

Percentage differences are shown for all other variables which are log transformed

Associations are adjusted for age (in quartiles), ethnic group, month of measurement, height, sex, an interaction between VO_2 max_ and sex and school (random effect). P-values are for interaction between VO_2 max_ and sex.

Supplementary Table 2: Associations between estimated VO_2 max_ and risk markers for type 2 diabetes and cardiovascular disease (differences per one unit increase in estimated VO_2 max_ in ml O_2_/min/kg) with adjustments for fat mass index: by ethnic group

| Risk markers (N = 1445) | Adjustments | % difference/difference* (95% CI) for a one IQR increase in estimated VO_2 max_ | | | | | | | | p(ethnic difference in association) |
| --- | --- | --- | --- | --- | --- | --- | --- | --- | --- | --- |
|  |  | White European (n=389) | | South Asian (n=373) | | Black African-Caribbean (n=346) | | Other (n=337) | |  |
| Insulin (mU/l) | Standard | -17.68 | (-23.57, -11.33) | -22.49 | (-28.41, -16.08) | -17.72 | (-24.40, -10.45) | -17.36 | (-23.87, -10.29) | 0.61 |
|  | Standard + FMI | -5.49 | (-11.73, 1.19) | -10.64 | (-16.93, -3.88) | -7.52 | (-14.38, -0.11) | -7.37 | (-14.04, -0.18) | 0.73 |
| HOMA Insulin resistance | Standard | -17.88 | (-23.80, -11.50) | -22.43 | (-28.40, -15.96) | -17.29 | (-24.05, -9.92) | -17.25 | (-23.81, -10.12) | 0.61 |
|  | Standard + FMI | -5.84 | (-12.12, 0.90) | -10.61 | (-16.97, -3.77) | -7.11 | (-14.08, 0.42) | -7.32 | (-14.06, -0.05) | 0.77 |
| HbA1c (%) | Standard | 0.00 | (-0.80, 0.82) | -0.98 | (-1.83, -0.12) | -1.16 | (-2.08, -0.24) | -0.28 | (-1.17, 0.62) | 0.16 |
|  | Standard + FMI | 0.24 | (-0.59, 1.07) | -0.73 | (-1.60, 0.15) | -0.96 | (-1.89, -0.03) | -0.08 | (-0.98, 0.83) | 0.16 |
| Glucose (mmol/l) | Standard | -1.59 | (-2.52, -0.64) | -0.69 | (-1.70, 0.33) | -0.83 | (-1.91, 0.26) | -0.71 | (-1.76, 0.34) | 0.51 |
|  | Standard + FMI | -1.24 | (-2.20, -0.28) | -0.32 | (-1.36, 0.72) | -0.54 | (-1.63, 0.57) | -0.43 | (-1.49, 0.64) | 0.52 |
| Urate (mmol/l) | Standard | -4.68 | (-7.69, -1.57) | -7.14 | (-10.27, -3.90) | -5.22 | (-8.63, -1.68) | -6.46 | (-9.71, -3.09) | 0.72 |
|  | Standard + FMI | -0.71 | (-3.77, 2.46) | -3.20 | (-6.39, 0.10) | -1.81 | (-5.23, 1.74) | -3.26 | (-6.52, 0.12) | 0.63 |
| C-reactive protein (mg/l) | Standard | -40.66 | (-49.58, -30.15) | -47.44 | (-55.83, -37.45) | -44.43 | (-53.89, -33.04) | -28.54 | (-40.30, -14.46) | 0.07 |
|  | Standard + FMI | -18.80 | (-29.98, -5.83) | -26.99 | (-37.68, -14.46) | -27.88 | (-39.01, -14.71) | -8.82 | (-22.46, 7.22) | 0.11 |
| Triglyceride (mmol/l) | Standard | -11.54 | (-15.73, -7.14) | -12.54 | (-16.96, -7.88) | -4.26 | (-9.42, 1.19) | -9.41 | (-14.13, -4.42) | 0.07 |
|  | Standard + FMI | -6.83 | (-11.20, -2.25) | -7.66 | (-12.28, -2.80) | 0.05 | (-5.24, 5.63) | -5.44 | (-10.28, -0.34) | 0.12 |
| HDL cholesterol (mmol/l) | Standard | 2.67 | (-0.08, 5.50) | 4.56 | (1.57, 7.64) | 4.68 | (1.48, 7.97) | 3.54 | (0.48, 6.69) | 0.78 |
|  | Standard + FMI | -0.52 | (-3.14, 2.18) | 1.21 | (-1.64, 4.14) | 1.93 | (-1.10, 5.06) | 0.88 | (-2.02, 3.88) | 0.67 |
| LDL cholesterol (mmol/l) | Standard | -5.84 | (-9.00, -2.57) | -4.68 | (-8.09, -1.15) | 1.36 | (-2.53, 5.41) | -3.41 | (-6.99, 0.30) | 0.03 |
|  | Standard + FMI | -4.15 | (-7.42, -0.77) | -2.84 | (-6.37, 0.82) | 2.92 | (-1.05, 7.05) | -1.97 | (-5.62, 1.81) | 0.04 |
| Systolic blood pressure (mmHg)* | Standard | -2.37 | (-3.69, -1.05) | -3.10 | (-4.52, -1.69) | -1.66 | (-3.17, -0.15) | -2.58 | (-4.04, -1.12) | 0.55 |
|  | Standard + FMI | -1.55 | (-2.89, -0.21) | -2.26 | (-3.69, -0.83) | -0.97 | (-2.48, 0.54) | -1.91 | (-3.37, -0.44) | 0.61 |
| Diastolic blood pressure (mmHg)* | Standard | -2.87 | (-4.06, -1.68) | -3.81 | (-5.08, -2.53) | -2.66 | (-4.02, -1.30) | -3.34 | (-4.65, -2.02) | 0.57 |
|  | Standard + FMI | -2.33 | (-3.54, -1.11) | -3.24 | (-4.54, -1.95) | -2.20 | (-3.57, -0.83) | -2.88 | (-4.21, -1.56) | 0.61 |

* Absolute differences are shown for untransformed variables

Percentage differences are shown for all other variables which are log transformed

Associations are adjusted for sex, age (in quartiles), ethnic group, month of measurement, height, an interaction between VO_2 max_ and ethnic group and school (random effect). P-values are for interaction between VO_2 max_ and ethnic group.

Supplementary Table 3: Associations between estimated VO_2 max_, physical activity and risk markers for type 2 diabetes and cardiovascular disease (differences per one IQR increase in estimated VO_2 max_ or counts), with adjustment for fat mass index

|  |  | % difference / difference* (95% CI) for a one IQR increase in estimated VO_2 max_ or counts, p-value | | | | | |
| --- | --- | --- | --- | --- | --- | --- | --- |
| Risk markers (N = 1083) | Variable | Standard adjustment | | | Standard + fat mass index adjustment | | |
| Insulin (mU/l) | Estimated VO_2 max_ | -18.11 | (-21.99, -14.04) | <0.0001 | -6.43 | (-10.67, -1.98) | 0.005 |
|  | Counts | -16.60 | (-21.34, -11.57) | <0.0001 | -7.68 | (-12.45, -2.65) | 0.003 |
| HOMA Insulin resistance | Estimated VO_2 max_ | -17.79 | (-21.67, -13.72) | <0.0001 | -6.23 | (-10.48, -1.77) | 0.01 |
|  | Counts | -16.28 | (-21.02, -11.25) | <0.0001 | -7.45 | (-12.23, -2.41) | 0.004 |
| HbA1c (%) | Estimated VO_2 max_ | -0.64 | (-1.17, -0.10) | 0.02 | -0.38 | (-0.95, 0.19) | 0.19 |
|  | Counts | -0.22 | (-0.86, 0.42) | 0.50 | 0.00 | (-0.65, 0.65) | 1.00 |
| Glucose (mmol/l) | Estimated VO_2 max_ | -0.95 | (-1.57, -0.33) | 0.003 | -0.64 | (-1.30, 0.03) | 0.06 |
|  | Counts | -0.12 | (-0.86, 0.63) | 0.76 | 0.18 | (-0.58, 0.94) | 0.64 |
| Urate (mmol/l) | Estimated VO_2 max_ | -5.61 | (-7.60, -3.58) | <0.0001 | -1.94 | (-4.06, 0.23) | 0.08 |
|  | Counts | -2.82 | (-5.26, -0.31) | 0.03 | 0.14 | (-2.32, 2.67) | 0.91 |
| C-reactive protein (mg/l) | Estimated VO_2 max_ | -43.95 | (-49.78, -37.44) | <0.0001 | -23.65 | (-31.22, -15.24) | <0.0001 |
|  | Counts | -28.20 | (-37.16, -17.97) | <0.0001 | -8.01 | (-18.39, 3.68) | 0.17 |
| Triglyceride (mmol/l) | Estimated VO_2 max_ | -10.38 | (-13.25, -7.41) | <0.0001 | -5.54 | (-8.65, -2.31) | <0.001 |
|  | Counts | -9.33 | (-12.79, -5.74) | <0.0001 | -5.52 | (-9.06, -1.85) | 0.004 |
| HDL cholesterol (mmol/l) | Estimated VO_2 max_ | 3.84 | (1.99, 5.73) | <0.0001 | 0.86 | (-0.99, 2.76) | 0.36 |
|  | Counts | 2.44 | (0.27, 4.67) | 0.03 | 0.23 | (-1.87, 2.37) | 0.83 |
| LDL cholesterol (mmol/l) | Estimated VO_2 max_ | -3.06 | (-5.20, -0.87) | 0.01 | -1.22 | (-3.53, 1.14) | 0.31 |
|  | Counts | -2.71 | (-5.23, -0.12) | 0.04 | -1.34 | (-3.93, 1.32) | 0.32 |
| Systolic blood pressure (mmHg)* | Estimated VO_2 max_ | -2.56 | (-3.42, -1.69) | <0.0001 | -1.74 | (-2.66, -0.83) | <0.001 |
|  | Counts | -0.37 | (-1.42, 0.67) | 0.48 | 0.41 | (-0.64, 1.45) | 0.45 |
| Diastolic blood pressure (mmHg)* | Estimated VO_2 max_ | -3.40 | (-4.17, -2.62) | <0.0001 | -2.90 | (-3.73, -2.08) | <0.0001 |
|  | Counts | -1.67 | (-2.62, -0.72) | <0.001 | -1.09 | (-2.05, -0.13) | 0.03 |

* Absolute differences are shown for untransformed variables

Percentage differences are shown for all other variables which are log transformed

Standard adjustment is for sex, age (in quartiles), ethnic group, month of measurement, height, and school (random effect)

Supplementary Table 4: Associations between estimated VO_2 max_ and risk markers for type 2 diabetes and cardiovascular disease (differences per one IQR increase in estimated VO_2 max_) by tertiles of physical activity counts

|  | % difference / difference* (95% CI) for a one IQR increase in estimated VO_2 max_ by tertiles of physical activity counts | | | | | | p (interaction) |
| --- | --- | --- | --- | --- | --- | --- | --- |
| Risk markers (N = 1083) | Lowest tertile of PA counts | | Middle tertile of PA counts | | Highest tertile of PA counts | |  |
| Insulin (mU/l) | -21.27 | (-27.32, -14.72) | -18.39 | (-24.97, -11.22) | -9.03 | (-15.84, -1.66) | 0.03 |
| HOMA-IR | -20.99 | (-27.06, -14.42) | -18.01 | (-24.61, -10.82) | -8.88 | (-15.70, -1.51) | 0.03 |
| HbA1c (%) | -1.31 | (-2.21, -0.41) | 0.15 | (-0.79, 1.11) | -0.72 | (-1.59, 0.16) | 0.08 |
| Glucose (mmol/l) | -1.26 | (-2.31, -0.21) | -0.83 | (-1.92, 0.28) | -0.73 | (-1.75, 0.30) | 0.77 |
| Urate (mmol/l) | -6.73 | (-10.03, -3.31) | -5.98 | (-9.45, -2.39) | -3.30 | (-6.61, 0.14) | 0.36 |
| CRP (mg/l) | -42.22 | (-52.00, -30.45) | -42.55 | (-52.65, -30.31) | -41.64 | (-51.24, -30.16) | 0.99 |
| Triglyceride (mmol/l) | -13.04 | (-17.63, -8.19) | -10.31 | (-15.26, -5.07) | -4.34 | (-9.25, 0.83) | 0.04 |
| HDL cholesterol (mmol/l) | 6.47 | (3.29, 9.74) | 1.84 | (-1.34, 5.12) | 2.51 | (-0.47, 5.57) | 0.08 |
| LDL cholesterol (mmol/l) | -5.08 | (-8.61, -1.42) | -0.96 | (-4.78, 3.02) | -1.59 | (-5.13, 2.08) | 0.23 |
| Systolic BP (mmHg)* | -3.15 | (-4.60, -1.71) | -1.83 | (-3.34, -0.31) | -2.54 | (-3.94, -1.13) | 0.47 |
| Diastolic BP (mmHg)* | -3.80 | (-5.10, -2.49) | -3.00 | (-4.37, -1.63) | -3.04 | (-4.31, -1.77) | 0.61 |

* Absolute differences are shown for untransformed variables

Percentage differences are shown for all other variables which are log transformed

Differences are adjusted for sex, age (quartiles), ethnic group, month, height, an interaction between estimated VO_2 max_ and tertiles of PA and school (random effect)

Supplementary Table 5: Ethnic differences in risk markers for type 2 diabetes and cardiovascular disease: effect of adjustment for estimated VO_2 max_ and physical activity counts

| Risk markers (N = 1083) | Adjustments | % difference / difference* (95% CI), p-value, % reduction in ethnic difference following adjustment for estimated VO_2 max_ | | | |
| --- | --- | --- | --- | --- | --- |
|  |  | South Asian - white European | | | |
| Insulin (mU/l) | Standard | 44.49 | (30.34, 60.18) | <0.0001 |  |
|  | Standard + VO_2 max_ | 41.15 | (27.68, 56.04) | <0.0001 | 7.5 |
|  | Standard + VO_2 max_ + counts | 38.24 | (25.09, 52.76) | <0.0001 | 14.0 |
| HOMA Insulin resistance | Standard | 44.03 | (30.00, 59.56) | <0.0001 |  |
|  | Standard + VO_2 max_ | 40.76 | (27.40, 55.53) | <0.0001 | 7.4 |
|  | Standard + VO_2 max_ + counts | 37.94 | (24.88, 52.36) | <0.0001 | 13.8 |
| HbA1c (%) | Standard | 1.66 | (0.56, 2.76) | 0.003 |  |
|  | Standard + VO_2 max_ | 1.58 | (0.49, 2.69) | 0.004 | 4.8 |
|  | Standard + VO_2 max_ + counts | 1.58 | (0.48, 2.68) | 0.005 | 4.8 |
| Glucose (mmol/l) | Standard | 1.60 | (0.32, 2.90) | 0.01 |  |
|  | Standard + VO_2 max_ | 1.49 | (0.22, 2.79) | 0.02 | 6.9 |
|  | Standard + VO_2 max_ + counts | 1.52 | (0.23, 2.82) | 0.02 | 5.0 |
| Urate (mmol/l) | Standard | 0.87 | (-3.43, 5.35) | 0.70 |  |
|  | Standard + VO_2 max_ | 0.18 | (-4.03, 4.56) | 0.94 | 79.3 |
|  | Standard + VO_2 max_ + counts | -0.01 | (-4.21, 4.38) | 1.00 | 101.1 |
| C-reactive protein (mg/l) | Standard | 20.11 | (-4.33, 50.81) | 0.11 |  |
|  | Standard + VO_2 max_ | 11.08 | (-10.93, 38.53) | 0.35 | 44.9 |
|  | Standard + VO_2 max_ + counts | 8.35 | (-13.14, 35.16) | 0.48 | 58.5 |
| Triglyceride (mmol/l) | Standard | 17.50 | (9.85, 25.68) | <0.0001 |  |
|  | Standard + VO_2 max_ | 15.96 | (8.54, 23.90) | <0.0001 | 8.8 |
|  | Standard + VO_2 max_ + counts | 14.73 | (7.39, 22.57) | <0.0001 | 15.8 |
| HDL cholesterol (mmol/l) | Standard | -5.76 | (-9.16, -2.24) | 0.002 |  |
|  | Standard + VO_2 max_ | -5.22 | (-8.63, -1.68) | 0.004 | 9.4 |
|  | Standard + VO_2 max_ + counts | -4.96 | (-8.40, -1.39) | 0.01 | 13.9 |
| LDL cholesterol (mmol/l) | Standard | 1.68 | (-2.74, 6.30) | 0.46 |  |
|  | Standard + VO_2 max_ | 1.29 | (-3.10, 5.89) | 0.57 | 23.2 |
|  | Standard + VO_2 max_ + counts | 1.04 | (-3.35, 5.64) | 0.65 | 38.1 |
| Systolic blood pressure (mmHg)* | Standard | -0.57 | (-2.38, 1.23) | 0.53 |  |
|  | Standard + VO_2 max_ | -0.88 | (-2.66, 0.89) | 0.33 | -54.4 |
|  | Standard + VO_2 max_ + counts | -0.83 | (-2.62, 0.95) | 0.36 | -45.6 |
| Diastolic blood pressure (mmHg)* | Standard | 1.62 | (-0.01, 3.26) | 0.05 |  |
|  | Standard + VO_2 max_ | 1.24 | (-0.35, 2.83) | 0.13 | 23.5 |
|  | Standard + VO_2 max_ + counts | 1.13 | (-0.46, 2.73) | 0.16 | 30.2 |

* Absolute differences are shown for untransformed variables

Percentage differences are shown for all other variables which are log transformed. Standard adjustment is for sex, age (in quartiles), ethnic group, month of measurement, height, and school (random effect).

Supplementary Figure 1: Mean fasting insulin by tertiles of physical activity and physical fitness


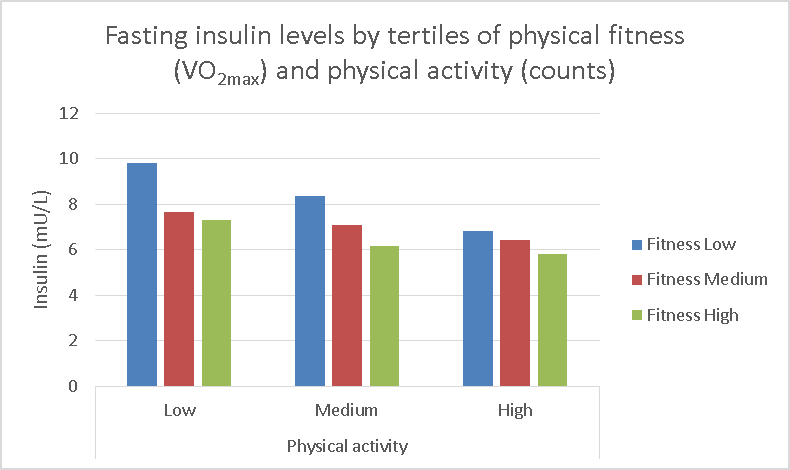


Geometric means are adjusted for sex, age (in quartiles), ethnic group, month of measurement, height, and school (random effect)
